# Trapped in my lungs and fighting a losing battle. A phenomenological study of patients living with chronic obstructive and pulmonary disease

**DOI:** 10.1111/scs.12713

**Published:** 2019-05-16

**Authors:** Hanneke van der Meide, Truus Teunissen, Leo H. Visser, Merel Visse

**Affiliations:** ^1^ University of Humanistic Studies Utrecht The Netherlands; ^2^ Tilburg University, Tilburg, Tranzo Scientific Center for Care and welfare The Netherlands; ^3^ Amsterdam UMC Amsterdam The Netherlands; ^4^ Elisabeth‐Tweesteden Hospital Tilburg The Netherlands

**Keywords:** phenomenology, COPD, breathlessness, lived experiences, lifeworld‐led health care

## Abstract

Chronic obstructive and pulmonary disease (COPD) has detrimental effects on individuals with the disease. COPD causes breathlessness, morbidity and associated psychosocial distress. This study was guided by the phenomenological question what is it like to have COPD and situated in Van Manen's phenomenology of practice. Experiential material was gathered through phenomenological interviews. Four themes emerged from the lived experiences of patients living with COPD: breath as a possibility; being vigilant; fighting a losing battle; and feeling isolated from others. For patients with COPD, breathing becomes ever‐present and shifts from the invisible background of daily living to the central activity around which everyday life is organised. COPD patients always monitor their own breath and scrutinise the environment on possible dangers that can affect their breathing. Whenever moving or being involved in an activity, a part of their mind is preoccupied with the breathing. Although COPD patients realise that no amount of good behaviour will matter and that the decline of their lungs is inevitable, they make every effort to take good care of their body. They anticipate and avoid triggers of breathlessness isolating them from social interactions and activities. The appearance of the body as a source of social embarrassment also has an isolating effect. This study shows that breathlessness is a constant horizon that frames the experience of COPD patients. It is a limiting factor and determines their entire life. A more profound understanding of these experiences in healthcare professionals will contribute to person‐centred care for COPD patients.

## Introduction

Trapped. That is what breathlessness feels like. Trapped in the web of uncertainty, bodily doubt, practical obstacles, and fear. The deepest fear you can think of. The fear of suffocation, of being unable to breathe, fear of collapsing, desaturated to the point of respiratory failure. (1, p. 109)

The phenomenology of breathlessness has vividly been expressed by philosopher Havi Carel who suffers from a respiratory illness herself. She notes that, phenomenologically speaking, breathlessness is remarkable in two intertwined ways: it is an overpowering sensation, to which we are deeply sensitive, but it is also behaviourally subtle, and often invisible to others (p. 109). Williams and Carel (2, p. 147) argue that breathlessness involves sensation, cognition and reasoning, none of which are reducible to the other, and it should therefore be approached and studied as an holistic experience rather than merely a bodily symptom. In line with this reasoning, our paper reports on a study into the lived experiences of patients with chronic obstructive pulmonary disease (COPD).

Chronic obstructive pulmonary disease is an umbrella term used to describe progressive obstructive lung diseases, including emphysema and chronic bronchitis. The disease is progressive and (currently) incurable. To date, only smoking cessation and supplementary oxygen therapy increase survival in patients 3. COPD is a major cause of morbidity and mortality and is predicted to become the third leading cause of death worldwide by 2020 3, 4. Dyspnoea, a feeling of breathlessness, is a common symptom, but often, COPD patients also suffer from disabling physical symptoms, comorbidity, anxiety and psychological distress. Moreover, COPD may have far‐reaching social consequences. The avoidance of triggers of breathlessness such as heat, cold, viral infections or smoke can isolate patients 5. Individuals with COPD may also be stigmatized due to the association of COPD with smoking. This might as well restrict COPD patients in their social interactions 6.

Areas of COPD research include the development and use of life‐changing treatments like pulmonary rehabilitation to provide a better and longer future for those diagnosed with COPD 7, 8. Another line of research is aimed at increasing the understanding of how damage occurs in the lungs to identify targets for new treatments that can stop the disease in its tracks 4, 9, 10. Qualitative studies on COPD patients show that the disease has detrimental effects on patients’ daily lives and causes disability 6, 11, 12, 13. Breathlessness is usually identified as the most troublesome symptom leading to panic and fear 14. If we want to understand the experience of COPD closely, we must attend to how it presents itself to those who live it. The aim of this study was to explore the daily experiences of COPD patients. A thorough understanding of patient's individual accounts is a very important process in developing the services for patients with COPD and supports healthcare professionals to become more attuned to the experiences that COPD patients may have in their daily live 15, 16. This is especially relevant because COPD is likely to increase in coming years due to higher smoking prevalence and ageing populations in many countries 17.

## Methods

### Phenomenological research design

This study was situated in Van Manen's phenomenology of practice that is a particular articulation of phenomenology referring to the kinds of inquiries that address and serve the practice of professional practitioners as well as the quotidian practices of everyday life (18, p. 15). A phenomenology of practice aims to ‘open up possibilities for creating formative relations between being and acting, between who we are and how we act, between thoughtfulness and tact’ (18, p. 69–70). The phenomenological question guiding this study was: What is the lived experience of COPD like? A phenomenological question explores human experience (a phenomenon) as it is lived through rather than how it can be conceptualised or theorised. Van Manen assumes that phenomenological inquiry cannot really be separated from the practice of writing because it is in the act of writing that insights emerge 19. Hence, this emphasis on writing is reflected in our research process.

### Recruitment and interviewing

The study was approved by the Medical Ethical Committee Brabant. Empirical material for our study consisted of phenomenological interviews. The Lung Foundation Netherlands spread a call for participation among its members.^1^ A rich variation in data is required for phenomenological research, and heterogeneous sampling is therefore recommended 20. The selection criterion was having COPD, regardless of the stage. We received 20 reactions from people that were interested to participate in our study. Nine people with COPD were interviewed. This number was gradually determined on the basis of the amount of experiential data as this is a critical condition for the possibility of proper phenomenological reflection and analysis 18. All participants provided written informed consent for the interview study. The interviews were conducted by the first two authors and a research assistant. On the participants’ request, all interviews took place at the homes of the participants. The interviews lasted between 33 and 84 minutes with an average of 63 minutes. The interviews were audio‐recorded.

In order to derive meaning from experiential material, it should be concrete, vivid and express descriptive detail 21. We knew that from our earlier (phenomenological) research experience, it is much easier for a person to tell *about* an experience than to tell an experience as lived through. We therefore drew up an interview guide with questions that asked for actual daily events inviting the participants to share a detailed experiential account of a moment in a particular place in time. Examples of the interview questions asked are as follows: Could you describe your morning routine? Can you describe a situation of your home environment and the role of COPD in this? Can you give an example of how you experience your body in public space? Can you give an example of a bad day? Can you give an example of a situation where you could concentrate on nothing but your body? Could you remember a moment when you forgot about your illness? During the unfolded conversations, we asked follow‐up questions such as: When was that, what happened, who were you with, how did you feel? The interviews were transcribed verbatim with all identifiable information removed from the transcripts.

### Data analysis and textual explication of the phenomenon

**Figure 1:**
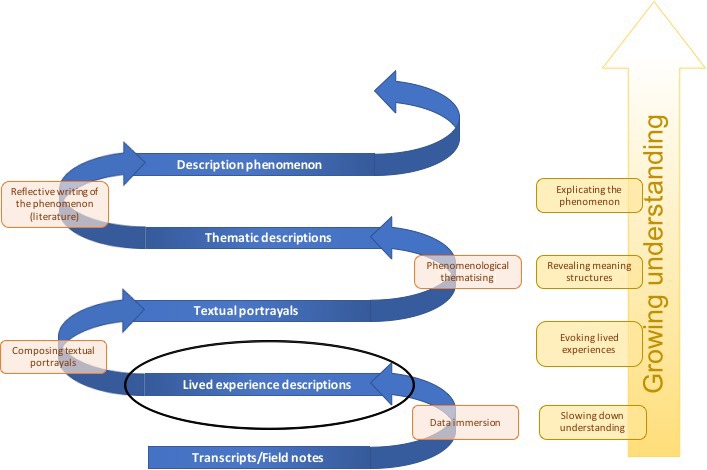
Phenomenological analysis.

Van Manen describes the phenomenological method not as a controlled set of procedures but as a way towards human understanding. Hence, he does not identify a series of steps that researchers have to go through during their research. Phenomenological analysis has been described as a state of active passivity 18, 20. There is an active search for meanings, but this is done from an open attitude of wonder. Figure 1 shows the reflective methods that were applied in this study. The following text and table 1 describe the analysis process in more detail.

The analysis was done by the first two authors as a joint process

**Figure 2:**
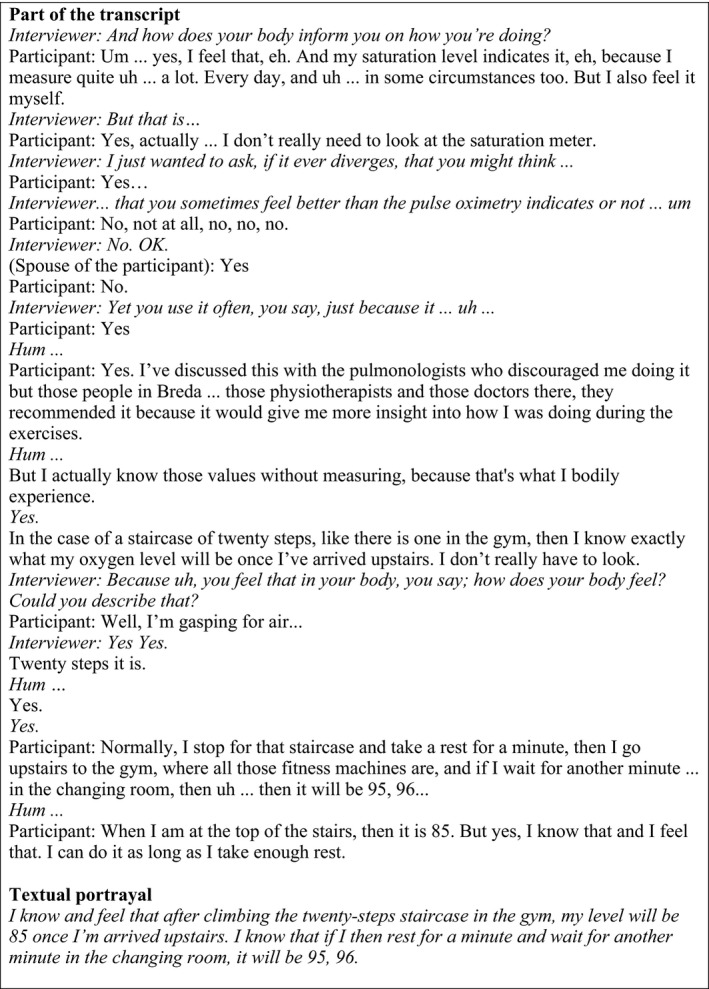
Example of the process of converting a transcript into a textual portrayal.

Immersion in lived experiences was pursued by reading the transcripts and listening to the audio files without doing anything actively immediately. In order to surface preunderstanding, thoughts, questions and comments were written in the margin and subsequently discussed. Then, concrete, lived experience descriptions that would form the basis of the analysis were identified. Other texts from the transcripts that were not suitable for phenomenological analysis such as extensive factual information, opinions and judgments were removed. The lived experience descriptions were used to compose textual portrayals.^2^ A textural portrayal was crafted from one transcript, based on one person's experience, and describes an aspect of the experience in an evocative language. Figure 2 provides an example and shows a portion of a transcript and the textual portrayal that emerged from this transcript. The actual words of the participants were used, but adjustments were made with regard to grammar and syntax. Polishing the lived experiences from the verbatim transcript increased the understanding in the researchers. Textual portrayals are also useful to convey meaning to the readers or hearers of the study. The central activities and the rational of moving from transcript to textual portrayal are described in Table 1. The textual portrayals formed the basis of a phenomenological thematisation that was aimed at revealing the structures of meaning in the experiences. The themes were identified and grouped with the aid of qualitative software atlas.ti. (version 1.0.49 Berlin, Germany). The use of qualitative software was helpful to order the large amount of data and to do a part of the analysis remotely. Theme analysis in phenomenological inquiry carries a different meaning from the way it is used in other qualitative research approaches. Phenomenological themes are not generalisations and are described by Van Manen as ‘knots in the web of our experiences, around which certain lived experiences are spun and thus lived through as meaningful wholes. Themes are the stars that make up the universes of meaning we live through’ 22. The themes were further finalised by means of reflective writing, aimed at disclosing the phenomenon. Literature was used in this phase to illuminate rather than impose meaning 23.

In the next section, the phenomenon of living with COPD will be explicated. The description of each theme starts with an experiential vignette that is composed of common fragments from verbatim data provided by participants during the interviews. The aim of these vignettes is to let the reader experience what one cannot know in an intellectual or cognitive sense 18. The singular form of the first person is used for the purpose of drawing the reader into the experience. The vignettes are followed by a phenomenological reflection in which textual portrayals are presented in italics.

**Table 1:** Reflective methods in phenomenological analysis

Reflective method	Central activities	‘Rational’
Immersion in lived experience	Reading interview transcripts Listening audio files Writing down our own thoughts Identifying lived experience descriptions	Suspending understanding Becoming familiar with the data Surfacing fore‐understanding Removal of the text that is not suitable for phenomenological analysis (e.g. opinions, factual information)
Composing textual portrayals	Editing of the actual content but not the phenomenological content (the meaning) Avoiding general statements and theoretical terms Focusing on the words and phrases that express meaning and the removal of unnecessary text Changing tense and pronouns if necessary	Textual portrayals are crafted from different parts of the verbatim transcribed interviews Ensure the story flows The aim is to convey ‘felt’ knowing that is difficult to describe The textual story serves as a methodological device for phenomenological analysis
Phenomenological thematisation	Wholistic reading of textual portrayals: focusing on the meaning of the portrayal as a whole Detailed reading of textual portrayals: searching for sentences and words that seemed to particularly reveal this meaning	The goal of thematisation is disclosing the meaning structure of the phenomenon as appears in the interviews The meaning structure shows possibilities of experiences
Reflective writing	Describing the themes with the aid of textual portrayals and phenomenological literature	Further explication of the phenomenon by a reflective process of writing and rewriting in which new insights may emerge. Literature is used to illuminate meanings: explicating the already but sometimes hidden meaning

## The experience of living with COPD

### Breath as a possibility

I wake up and the first thing I do is coughing. If my coughing is effective in clearing mucus out of my lungs, I know it will be a good day because I will need less energy that day to cough away my mucus. If the mucus remains, I have to take it easy. Once I get up, I become aware of my breathing and as soon as I take a few steps, I'm out of breath. Despite being inside the walls of my bedroom, I already know what the weather is like. If I feel my lungs, as if something is constantly itching them, it's raining or foggy outside. If I can breathe reasonably, the air should be dry. One of the most difficult things with COPD is drying off after showering. Taking a shower is already quite a task which I certainly cannot do every day but drying off is especially challenging. Going back and forth with a towel, bending over to reach my knees and feet and stretching my arms to dry my hair makes me gasping for air. I have to rest regularly to catch my breath again. Then I sit down on the toilet in my bathrobe and wait until I've dried up. This saves me energy and leaves me enough breath to get dressed.

While breathing goes well, breathing forms a prereflective essence of bodyliness. It is described as a typically unconscious and forgotten fact of life in its reality as a finite number of breaths and breathtaking moments 24. It is only when their breathing falters and they have to gasp for air that people notice that breath is a condition of possibility, for any action and for life in gteneral. For the participants in this study, breathing is ever‐present and has become an explicit process. Instead of the invisible background of daily living, breathing has become the central activity around which everyday life is organised. The act of breathing and the modulation of breath have become explicit primary tasks and challenges. Participants state that breathlessness may be exacerbated by environmental triggers such as excessive heat or cold, fine dust or smoke. ‘Shortness of breath can suddenly overtake me. Outside, for example, if it's raining cats and dogs. But even if the weather is fine like last week, it may happen. I went out for a walk and I already had to turn back at the corner of the street because of all those exhaust gases from the cars on the road’.

Participants experience breathlessness as overwhelming and compelling. If their breathing falters, their body has to slow down or take rest immediately. The participants felt bodily limitation almost continuously because breathing has become noticeable in every movement. ‘When you just rang the doorbell, I jumped up and walked to the door, but I was immediately out of breath. I really have to think about ‘slowing down’. I usually forget as I'm used to do things quickly. I've always worked on the market, you know’. The compelling character might force the participants to change their bodily habits. Brisk movements, for example, have to be censored. In some situations and on a whim however, the participants fall back into what they were used to. Many actions, particularly everyday routine actions, are prereflective: they are the product of habit rather than conscious reflection. COPD forces the participants to change their habits like consciously planning a trip upstairs and to prepare the meal in advance so that one not has to do it all at once at dinnertime.

### Being vigilant

I'm always on my guard when we are outside the house. When we are in a restaurant or at a party, I always make sure that I don't sit in a drift. We almost never go out for dinner because of the risk. We avoid large companies as much as possible. When we receive an invitation for a party, I always inquire about the location and how many people there will be. Sometimes we cannot avoid such things and then we go along very briefly. I am very careful about catching a cold because those times that I was in the hospital, it all started off with a cold. When I'm biking I'm always aware of closing my mouth since catching a cold may have disastrous consequences. Bacteria are creepy and may lead to serious infections which I want to avoid at all costs.

To breathe is to live, and without breath, there is no life. The participants tell that they are always on their guard. Monitoring their own breath and scrutinising the environment on possible dangers that may affect breathing have become central activities in their daily life. The participants are always aware of their actual oxygen flow rate, and the pulse oximetry, a noninvasive medical technology, is important for many of them because it discloses the inner aspect of their body. It plays a mediating role in their bodily relation and assists them in shaping everyday life. It also provides them insight into their bodily possibilities and limits. It may explain why they are still tired after a full night of sleep. It tells them the development of their bodily functioning: Is there progress or decline? It may also help them in their contact with healthcare professionals. But perhaps most importantly, it can provide them with the feeling of rest and certainty. It is, as it were, an extra certainty because even without the pulse oximetry they know what their oxygen level is like. ‘I know and feel that after climbing the twenty‐steps staircase in the gym my level will be 85 once I'm arrived upstairs. I know that if I then rest for a minute and wait for another minute in the changing room, it will be 95, 96’.

The sense of calm is of great importance for the participants. Whenever moving or being involved in an activity, a part of the mind of the participant is preoccupied with the breathing. ‘A few days ago I was really scared when my husband wanted to have sex with me. I was afraid that my body was not able to. So I was very cautious and completely exhausted afterwards. But yeah, it sometimes has to be done as I cannot always refuse. I enjoyed it but I was also very preoccupied with my body’. Because of this vigilant attitude, participants calculate and consider the physicality of every action.

### Fighting a losing battle

I force myself every day to move. I go to the gym, three times a week. I always go, even if I'm not in the mood. It's something that I have to because I need to build up my muscle mass. On the other days, I do the groceries or I go to a meeting by bike. I've had pneumonia a few times and got hospitalized. After such an occurrence my condition is each time so deteriorated that I have to start exercising from scratch again. It's tough to rebuild everything again and again, thereby knowing that I will never become the same as before and that my body will hit me again in the foreseeable future. Even if I succeed in coming close to my previous level of condition, I always have to give up on something. But I just go on, I have no choice. If I do nothing, my body will deteriorate even faster. This motivates me to keep on fighting, even though I realize I will never win. No matter how hard I try, my lungs will never get better.

The daily experiences of the participants reflect a vicious circle; acute breathlessness must be avoided, leading to restricted activity, which in turn result in further deconditioning, which will cause the breathlessness to increase as fitness reduces. Our interviews show that social activities in particular are restricted and that physical activities become more important. The participants know and feel that their body is deteriorating. They also know that if they do nothing, this decline will go even faster. Out of necessity and self‐protection, the participants work hard to keep their body in shape and to not lose more muscle mass. The life of the participants is dominated by the concern for their own body. Rather than perceiving this as negative, it provides them a purpose in life. It contributes to the well‐being of the participants by enabling them to structure their day and providing a good feeling in the short term although the entire effort is at the same time experienced as fighting a losing battle.

An inevitable relapse or hospitalisation that prevents the participant from moving temporarily or to a very limited extent may mean that the hard‐fought condition is lost very quickly. The decline of lung function is out of control because COPD is stronger than the body, and stronger than the will. The participants realise that no amount of good behaviour will matter. Yet the motivation remains, even if their body fails on them. ‘My legs are aching at the moment. I feel my muscles in my legs as if they are playing on a pipe organ. Over and over again my body gets affected and confronting me with something I should deal with. But I remain active until I cannot do anything anymore. Because if I don't, my body will let me down and my life will lose meaning’.

### Feeling isolated from others

My sister lives in Amsterdam where they have a car‐free city policy. First I could park my car easily but that is no longer possible. I hardly ever see her again. Walking from the parking lot or the nearest public transport stop is simply too far. I could go with such a special taxi for disabled people but therefore I have to call and make an appointment. I've never done something like that in my life. I don't want to be dependent on a taxi driver. I'd really like to go to Enschede as well. There is an interesting museum that I'd love to visit. I'd like to see a painting that has always fascinated me. I don't go because I'm afraid of the distance that I have to walk and I'm not sure whether there are sufficient facilities. I have to urinate often because of the coughing that I cannot control and moreover, the coughing makes me uncomfortable. It is often accompanied by a lot of noise, mucus and can also look scary. When I am short of breath, I see people looking around me and wondering if things are going well. I rather stay home, where I can be who I am.

The participants have become housebound because they need to anticipate and avoid triggers of breathlessness. This avoidance isolates them from locations and activities. There can be real obstacles to participate, but often, it concerns possible obstacles and expectations. The participants are nervous about leaving the house and going into the unknown. Former experiences may play a role in this. The feeling of slowing the others down, for example, as actions become out of step with other's actions. COPD is also isolating in the sense that it is an indivisible experience that is difficult to share even with people close by, such as the partner or children. ‘A few years ago, my husband didn't see how bad I was. Yes he said that I had to take it easy but I felt that in is mind he was thinking something differently. For him it is also difficult because I always could do everything. Although he was with me in the hospital attending all the consultations, he didn't get the message. Last Sunday he had to help me with making the bed and he saw me panting. I then saw from the look in his eyes that he know realizes how serious it is’.

The vignette stresses these feelings of self‐consciousness and awkwardness. A body that coughs up mucus is difficult to ignore, and participants experience fear of offending others. The uncertainty of how the body will behave makes social interaction scary and for some something to be avoided. The appearance of the body in socially unacceptable ways draws attention to the person's unpredictable body and detracts from enjoyable participation in activities 12. ‘I have to cough when I get mucus. That's what I'm ashamed of, for that coughing. I went to the mall yesterday and I really had to cough. I felt stuffy and it didn't go away with simply coughing. So I coughed hard and people were looking at me. I knew that I had to cough even stronger but I didn't dare because I was afraid that people would call the emergency number’.

## Discussion

Healthy people are embodied in such a way that they are un‐preoccupied with their physical condition 25. Our study shows that breathlessness is a constant horizon that frames the experience of COPD patients. A part of their mind is continually preoccupied with breathing. The body with COPD determines possibilities and delineates with extreme clarity what one is and is not permitted to do and to be (1, p. 110).

In the following, we will discuss the implications of our findings using the lifeworld approach, a conceptual framework that articulates an approach to care with well‐being as focus 16. The lifeworld is an experienced world of meaning and refers to the qualitative dimension of human living. The lifeworld takes existential well‐being rather than health as a focus in situations of illness and vulnerability. From a lifeworld perspective, well‐being is always seen in relation to suffering. Galvin and Todres 16 argue that practitioners require a view and understanding of both. Studying possibilities of well‐being in COPD, which will only deteriorate from a biomedical perspective, offers healthcare practitioners directions for care. Also, a meaningful understanding of suffering can provide a human capacity for care and may entail empathic power.

Our phenomenological perspective on the experiences of COPD patients has disclosed dimensions of both suffering and (possibilities of) well‐being. A COPD patient may suffer on one dimension of the lifeworld without having to suffer on the other dimensions. Our study discloses a suffering experience in the spatial dimension of the lifeworld. The physical boundaries of COPD patients are diminished, and a great part of their life is limited and determined by their lung capacity. They are trapped into their lungs. Similar experiences of suffering in the spatial dimensions are described in the literature. Gullick and Stainton 5, for example, state that people with COPD and their close family members live within a shrinking lifeworld. Williams et al. 26 describe this experience as like living within a ‘stagnant pool’. The participants in our study spent their time mostly at home. Our interviews show that suffering in the spatial dimension is closely related to the experience of interpersonal suffering. The participants in this study called sputum production, uncontrolled coughing and urinary urgency reasons to stay home. COPD may display the body in socially unacceptable ways. Home is described as the place ‘where we can be what we are’ (22, p. 102) without being scrutinised. Hence, the social consequences of the disease are far‐reaching. The participants experience isolation from others, and their sense of belonging is ruptured.

The COPD patients in our study experience good days and bad days, and this means that planned activities often need to be adjusted or cancelled. Despite the uncertainty, there is a certain rhythm. Breathing is often worse in the mornings, coinciding with the need to clear sputum and the need to attend to morning rituals. This temporal dimension has also been stressed in a review on qualitative research literature on the experience of COPD for the patient and family 12. Despite the experience of ‘no respite’ and ‘fighting a losing battle’, the people in our study experience a sense of future and a meaningful purpose in life. Heidegger 27 characterises projection as an important feature of human being. Taking care of their body is central in the ‘new’ life project of COPD patients. Being engaged in voluntary work or being involved in the growing up of a grandchild are other examples that offers the participants of this study ‘an invitation into a welcoming future’ (16, p. 83).

It should be noted that in our interviews the experiences of guilt and own responsibility or trends like self‐management of COPD were not addressed. We did not bring forward these topics ourselves because of our phenomenological and non‐judgmental attitude. What people discussed on their own initiative were explanations for having COPD such as their former working conditions. They were all convinced that smoking could not be the only reason. This corresponds to the research of Hansen et al. 28 who studied how people with COPD account for their illness. This has relevance for the development of (smoking cessation) interventions for people with COPD and how healthcare professionals should approach this group of patients. It should also be noted that all people in our study had stopped smoking. This does not apply to all COPD patients, and it should be further examined whether the experience of taking care of the body is representative of living with COPD. With regard to the experience of isolation, it should be noted that the participants in our study were in their sixties and seventies. People of this age in general turn more inward. Nevertheless, depression and anxiety are common in people with COPD 12. Further research should focus on a deeper understanding of spatial and interpersonal suffering in COPD. Although greater spatial mobility may not possible, a sensitivity of the healthcare professional may help to reduce the person's sense of imprisonment. A phenomenology of practice is not focused on ‘how to act’, and it does not aim for technicalities and instrumentalities but aims for ‘nurturing a measure of thoughtfulness and tact in the practice of professions and in everyday life’ (18, p. 31). A phenomenological perspective honours human experience in its complexity without simply dichotomising the experience as either psychological or physical. This is important because there exist a large differ how practitioners think about disease and how patient experience their illness 29. The understanding gained from this study may support professionals to building trust and enhance well‐being in the intersubjective dimension. Although outside of the scope of this paper, there are examples of healthcare initiatives, that explicitly focus on this 30, 31. COPD patients usually have long‐lasting contact with their care professionals, creating possibilities for true relational partnerships. Our study is meant to sensitise professionals for what is at stake from the perspective of a COPD patient. It also invites them to further explore the lifeworld and life projects of each individual patient which are the conditions for person‐centred care.

There were some limitations to this study. First, the sample size for this study was small, but the interviews provided rich experiential data. In a phenomenological study, increasing understanding is a more important concern than the generalisability of the findings. Second, the focus of the study was how COPD patients experience their daily life and the data covered therefore a wide range of subjects. A focus on a more limited phenomenon would probably have led to more depth. Third, participants were only interviewed once. A follow‐up interview would have given the opportunity to further explore the experiences discussed.

We want to conclude this paper with reflecting on the quality of our study. Van Manen 18 identifies 6 criteria for ‘strong’ phenomenological writing. *Heuristic attentiveness* refers to the sense of wonder induced by the text and was mainly pursued in the result section in which lived experience descriptions are alternated with phenomenological reflection. The principle of *descriptive richness* is met by presenting concrete experiential (narrative) lifeworld material by means of textual portrayals. The four reflective methods have been deployed to gain *interpretive depth*. Also, the analysis process has been described in detail to demonstrate the soundness of the interpretative process and enhance validity (18, p. 348). This study was strongly rooted in the phenomenology of practice enhancing our phenomenological sensibility and *rigour* of the study. The experiential vignettes and textual portrayals are examples of how the principles of *strongly embedded meaning* and *experiential awakening* were taken into account. *Inceptual epiphany*, the possibility of deeper and original insight, was pursued in the discussion by using phenomenological literature and Galvin & Todres’ lattices of well‐being and suffering.

## Author contributions

The first, third and fourth authors were responsible for the research design and obtaining funding. The first and second authors conducted the interviews and analysis. The first author was responsible for the drafting of the manuscript, while the second, third and fourth authors commented on the various drafts, finally leading to this article.

## Ethical approval

The Medical Ethical Committee Brabant approved all study procedures.

## Funding

This work was supported by the National MS Foundation.

## Notes

1 As a patient's association, The Lung Foundation Netherlands promotes the interests of people with chronic lung disease through the entire country. They provide information, advice and experience. http://research.longfonds.nl/lung-foundation-netherlands.

2 With ‘textual portrayals’, we refer to a similar process of rewriting experiential descriptions that is described by Van Manen 18 as writing anecdotes and by Crowther et al. 32 as crafting stories. ‘Anecdote’ can easily be confused with questionable factual empirical accounts. ‘Story’ may suggest that the experience is a completed whole and has a beginning and end. We therefore opted for the term ‘textual portrayal’ as it was more suitable for our purpose.
